# Promising Tools to Facilitate the Implementation of TDM of Biologics in Clinical Practice

**DOI:** 10.3390/jcm11113011

**Published:** 2022-05-26

**Authors:** Rani Soenen, Christophe Stove, Alessio Capobianco, Hanne De Schutter, Marie Dobbelaere, Tahmina Mahjor, Merel Follens, Jo Lambert, Lynda Grine

**Affiliations:** 1 Department of Dermatology, Ghent University Hospital, 9000 Ghent, Belgium; rani.soenen@uzgent.be (R.S.); lynda.grine@gmail.com (L.G.); 2 Dermatology Research Unit, University Ghent, 9000 Ghent, Belgium; alessio.capobianco@ugent.be (A.C.); hanne.deschutter@ugent.be (H.D.S.); marie.dobbelaere@ugent.be (M.D.); tahmina.mahjor@ugent.be (T.M.); merel.follens@ugent.be (M.F.); 3 Department of Pharmaceutical and Pharmacological Sciences, KU Leuven, 3000 Leuven, Belgium; 4 Department of Bioanalysis, Ghent University Hospital, 9000 Ghent, Belgium; christophe.stove@ugent.be

**Keywords:** psoriasis, biologics, therapeutic drug monitoring, lateral flow testing, microsampling

## Abstract

Therapeutic drug monitoring (TDM) of biologics—encompassing the measurement of (trough) concentrations and anti-drug antibodies—is emerging as a valuable tool for clinical decision making. While this strategy needs further validation, attention on its implementation into the clinic is warranted. Rapid testing and easy sampling are key to its implementation. Here, we aimed to evaluate the feasibility and volunteers’ perception of home microsampling for quantification of adalimumab (ADM) concentrations in psoriasis patients. In addition, we compared lateral flow testing (LFT) with enzyme-linked immunosorbent assay (ELISA). Patients participating in the SUPRA-A study (clinicaltrials.gov NCT04028713) were asked to participate in a substudy where volumetric absorptive microsampling (VAMS) was performed at home. At three time points, whole blood and corresponding serum samples were collected for ADM measurement using an in-house ELISA. In addition, the patients’ perspective on microsampling was evaluated via a questionnaire. LFT-obtained ADM concentrations agreed very well with ELISA results (Pearson’s correlation = 0.95 and R^2^ = 0.89). ADM concentrations determined in both capillary (via finger prick) and corresponding venous blood VAMS samples correlated strongly with serum concentrations (Pearson’s correlation = 0.87). Our preliminary data (n = 7) on rapid testing and home-based microsampling are considered promising with regard to TDM implementation for adalimumab, warranting further research.

## 1. Introduction

The management of patients with moderate-to-severe psoriasis has changed dramatically over the past years and has greatly benefited from the use of biologics, monoclonal antibodies targeting specific components of the immune system [1]. The first class of biologics comprises anti-tumor necrosis factor (anti-TNF) agents, including infliximab, adalimumab (ADM), etanercept and golimumab, which remain widely used in psoriasis, in addition to psoriatic arthritis and inflammatory bowel disease [2]. Although the efficacy is superior compared to conventional systemic treatments such as methotrexate [3], real-world evidence on effectiveness revealed primary and secondary non-responders [4,5,6]. Primary non-responders are considered patients who do not respond or insufficiently respond to the biologic, whereas secondary non-responders include patients who responded well initially, yet lose clinical response over time. A variety of reasons exist for these observations, but the exact mechanisms remain to be unraveled [7].

**Figure 1:**
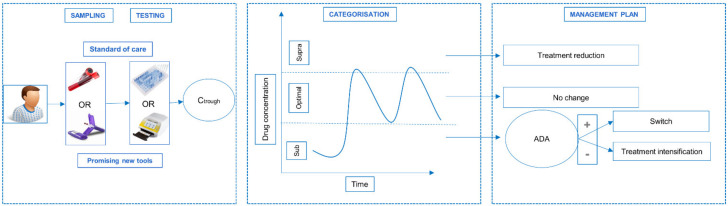
Concept of therapeutic drug monitoring. Therapeutic drug monitoring as proposed in the SUPRA-A trial: blood from the patient is sampled at trough as either whole blood (venipuncture or finger prick). Adalimumab (ADM) concentrations can then be quantified by ELISA or lateral flow testing (LFT). Based on the therapeutic window of ADM (dotted horizontal lines in graph) [8,9], the patients’ trough levels can be categorized into three categories: supratherapeutic (above upper threshold of window), optimal (within window), or subtherapeutic (below lower threshold of window). Depending on the categorization, a management plan is set up by the physician and the dose administration can be adapted. In case of subtherapeutic levels, additional testing of immunogenicity may be required to detect anti-drug antibodies (ADA). If ADA-negative, a dose increase (intensification) may be needed. If ADAs are present, a treatment switch is recommended. Figure adapted from Research Foundation—Flanders (FWO) grant proposal (T003218N). Abbreviations: ADA—Anti-Drug Antibodies; Ctrough—serum trough concentration (i.e., drug concentration right before the next drug administration.

One explanation entails drug exposure, which is usually measured through the trough (i.e., right before the next drug administration) concentrations in serum or plasma. To this end, a therapeutic window may be defined, making it possible to categorize patients as ‘underexposed’ or ‘overexposed’ (Figure 1).

The use of the concept of a therapeutic window, and corresponding adaptation of a dosing regimen to ensure that the concentrations of a drug lie within this window, is coined ‘therapeutic drug monitoring’ (TDM). TDM already found its way in the field of treatment of inflammatory bowel disease with anti-TNF agents [10]. Here, windows have been defined for infliximab, vedolizumab, golimumab, and ADM [8,9,11]. Interestingly, the definition of such windows heavily depend on several factors, including the timing of sampling (induction versus maintenance phase), the quantification assay but also the clinical outcome and of course the disease [10]. To illustrate the latter, Juncadella et al. evaluated ADM concentrations in light of several objective therapeutic outcomes: ADM levels of 11.8, 12.0, and 12.2 μg/mL were associated with biochemical, endoscopic or histological remission in patients with Crohn’s disease; respectively [11]. For ulcerative colitis, levels of 10.5, 16.2, and 16.2 μg/mL of ADM were defined as threshold for the respective outcomes. Endoscopic and histological remission required minimum levels of 12 and 12.2 μg/mL of ADM, respectively. Similar observations were made for infliximab [12]. In 2015, we defined a window for ADM in psoriasis of 3.51–7.0 mg/L associating with an optimal clinical effect, which was later confirmed by Wilkinson and colleagues in a larger cohort [8,9]. Currently, the use of TDM in patients with overexposure to ADM (supratherapeutic levels) is being investigated in the SUPRA-A trial (recruitment ongoing; clinicaltrials.gov NCT04028713) [13].

However, for TDM to be implemented in clinical practice, several criteria need to be met: first, a therapeutic window or target concentration should be defined based on a dose–response relationship, linking measurable drug concentrations (typically in serum) to a clinical therapeutic efficacy. In addition, serum concentrations should be available rapidly, in case of clinical flares, in order to propose dose adjustments, and, even more, should be obtained with relative ease for patient, clinician and lab. Currently, TDM of biologics is generally performed in serum as the sample matrix, requiring sampling to be done by a healthcare professional and the patient to leave their home. Not only is this time-consuming for the patient, but during the initial COVID-19 pandemic lockdown, nearly impossible [14]. Hence, alternative sampling methods that can be executed in the comfort of the patients’ homes have become more relevant. Sampling via dried blood spots (DBS) has long been a standard method of microsampling, with various studies supporting its feasibility in different patient populations [15,16,17,18,19]. It has numerous advantages, including the collection of microvolumes of equal to or less than 20 μL, which is more suitable for reduced blood flow or when handling infants. Moreover, storage and transfer of DBS does not require freezing, making it suitable for various settings (including patient homes or remote locations). Another asset is the simplicity of collection, omitting centrifugation or wet volume transfers and thus biohazardous handling. Lastly, its costs are much lower regarding transport and storage, making it an attractive option for large trials or—when needed regularly—in patient management.

Despite being a relatively robust sampling method for routine drug monitoring, the implementation of dried blood microsampling is hampered by a few issues. First, since whole blood is used as a source, the presence of haematocrit impacts the viscosity of the sample and thus its spreading on filter paper. This may pose an issue as, for a given volume applied on filter paper, blood with a high haematocrit will yield smaller-sized DBS than blood with a low haematocrit. Consequently, when using a partial-punch approach, the blood volume contained within this punch -and hence also the concentration derived for a compound- will differ. This is also referred to as the haematocrit effect [20,21].

Second, the volume of blood that is collected is not easily controlled. However, recently several devices have been developed that allow volumetric collection of blood from a finger prick [20,21]. One of these newer technologies is called volumetric absorptive microsampling (VAMS), in which a polymeric tip wicks up a fixed volume of blood (e.g., 10, 20 or 30 microliters), irrespective of the hematocrit [20,22]. The use of VAMS for various drugs has been tested and was found to be a reliable sampling method, also for the determination of larger proteins such as monoclonal antibodies [16,23].

After sample collection, the sample still needs to be assessed. Until now, in clinical laboratory settings, the measurement of biologics is predominantly performed by means of enzyme-linked immunosorbent assay (ELISA). ELISAs are antigen-specific, provide quantifiable results, and can easily be implemented in clinical laboratories (in essence only requiring an absorbance microplate reader). On a downside, the execution is rather time-consuming (impacting the turn-over time) and it is rather suitable for high-throughput measurement; i.e., to be cost-effective, multiple samples need to be analyzed simultaneously. Only if sufficient patients in a short time window need to be sampled, ELISA is cost-efficient, which may induce long waiting times to meet the serum sample number. To this end, alternative assays were developed, such as ‘rapid’ testing via lateral flow testing (LFT) assays. LFT has gained massive use amongst the general public since the COVID-19 pandemic [24]. In the field of TDM, LFT for the measurement of ADM and anti-ADM antibodies has been developed as well [25]. However, the use of such assays has remained limited to the clinical setting, due to the requirement of serum or plasma as a matrix and a specific reader.

Although recent advances in the microsampling and rapid testing field allow for several limitations to be tackled, this is yet to be shown for TDM of ADM in the psoriasis population. Here, we report on a preliminary evaluation of the use of rapid testing and home sampling by patients participating in a study assessing non-inferiority of a TDM-based dose reduction strategy for ADM in psoriasis (Soenen et al.; unpublished). In this study, two promising tools to facilitate TDM, self-sampling at home and rapid testing, were evaluated.

## 2. Materials and Methods

### 2.1. Study Design and Data Collection

Subjects were recruited from the SUPRA-A trial (NCT04028713) with ethical approval from the Ethics Committee of Ghent University Hospital in Belgium (EudraCT 2019-001918-42). All participating subjects provided written informed consent. The study was conducted in accordance with the Declaration of Helsinki. At the time of writing, the study had not been completed yet.

In short, patients with confirmed supratherapeutic ADM concentration were randomized 1:1 to a control or intervention arm, the latter implying a lengthening of the dose interval (once every 3 weeks instead of bi-weekly). If the therapeutic response remained stable in the intervention arm, the dose reduction could be extended to every 4 weeks. All patients were asked to participate in a substudy in which the use of VAMS was evaluated as an alternative sampling technique for quantification of ADM.

Study data were collected and managed using REDCap (Research Electronic Data Capture) hosted at Ghent University Hospital [26,27].

### 2.2. Sample Collection, Transportation, Preparation and Storage

On day 0, patients received a short training by an instructed nurse as well as written instructions on home sampling (Supplementary Figure S1). The first sampling was performed under supervision of the trained nurse in the hospital. Afterwards, patients were asked to self-sample at 9 pre-defined time points (day 3, 5, 7, 14, 21, 28, 35, 42, and 49) using a VAMS device, marketed as Mitra^®^ (Neoteryx LLC, Torrance, CA, USA). In short, the device consists of a plastic handler with an absorbent polymeric tip attached to it, which absorbs a precise volume of blood (20 μL), after a finger prick was performed with a 1.8 mm safety lancet (Novolab, Ergolance Blue 25G, Geraardsbergen, Belgium). For each time point, patients received a home sampling kit containing the following materials to perform home sampling (in duplicate, if needed): lancet, sampling cartridge, cotton ball, adhesive bandage and a prepaid shipping envelope with desiccant. At each time point, 2 VAMS samples were collected by the patients (biological replicates). The VAMS samples were sent by postal service under ambient conditions to the Dermatology Research Unit of Ghent University Hospital for processing. Upon receipt, the tips were first visually inspected for absorption efficiency [28]; i.e., if white spots were still visible we assumed less than 20 μL was absorbed and this was noted for each individual sample. Next, the VAMS tips were transferred to 1.5 mL Eppendorfs with a forceps and 480 μL superblock PBS buffer (ThermoFisher; Waltham, MA, USA) was added. Following an incubation of 1 h at 21 °C at 300 RPM (Eppendorf ThermoMixer C, Hamburg, Germany), each vial was shortly vortexed and tips were removed with cleaned forceps. Extracts were stored at −20 °C until analysis. Overall, extraction was done within 4 days after sampling (ranging from 1 day until 10 days).

**Figure 2:**
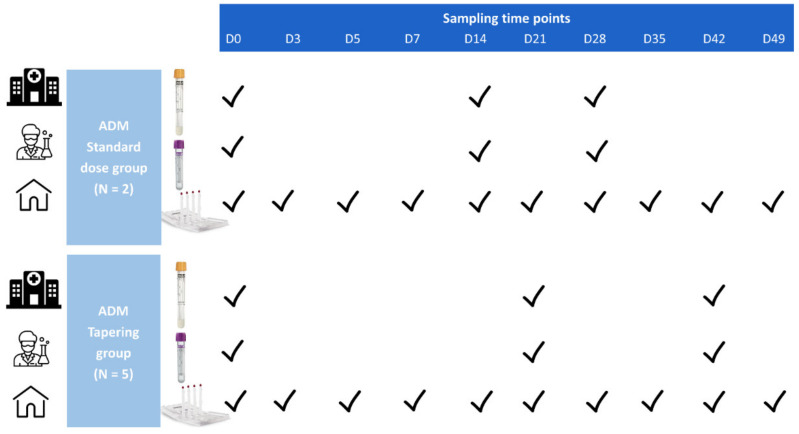
Overview of sampling time points in the substudy of the SUPRA-A trial. Patients were asked to self-sample at 10 predefined time points (day 0 (after training and under supervision of nurse), 3, 5, 7, 14, 21, 28, 35, 42, and 49) using a VAMS device, marketed as Mitra^®^ (Neoteryx LLC, Torrance, CA, USA). At day 0, day 14 and day 28 or day 0, day 21 and day 42 whole blood and serum were collected simultaneously, allowing comparison of the different matrices, i.e., capillary VAMS, venous VAMS and serum (gold standard) samples. Abbreviations: D—Day; N—Number of patients.

In addition, at 3 predefined time points (day 0, day 14 and day 28 or day 0, day 21 and day 42, depending on the randomization arm), whole blood and serum were collected simultaneously, allowing comparison of the different matrices, i.e., capillary VAMS samples (obtained following finger prick), venous VAMS samples and serum (gold standard) samples. Venous VAMS samples were obtained by a trained nurse by touching the surface of ethylenediaminetetraacetic acid (EDTA)-anticoagulated venous whole blood with a VAMS tip. The serum was centrifuged for 10 min at 252× *g* at room temperature (Eppendorf centrifuge 5804, Germany), after an incubation time of maximum 24 h. Serum was preserved at minimally −20 °C for a median time of 185 days until analysis. An overview of the sampling time points is depicted in Figure 2.

### 2.3. Extraction Efficiency of Adalimumab in Volumetric Absorptive Microsamples

The extraction efficiency of ADM from VAMS tips was evaluated by spiking the blood of healthy volunteers with known ADM concentrations, 0–1–5–10 and 20 μg/mL. After drying for a minimum of 24 h, VAMS tips were extracted and extracts were stored at −20 °C until analysis.

### 2.4. Quantification of Adalimumab Concentrations

#### 2.4.1. Rapid Testing with Lateral Flow Technique

ADM serum levels were measured using RIDA^®^QUICK ADM Monitoring lateral flow assay (R-Biopharm AG, Darmstadt, Germany), according to the manufacturer’s instructions. In short, after a 5 min pre-incubation of 1:500 diluted sample with “Solution A” and “Solution B” and 15 min developing time on the lateral flow strip, the lateral flow strips were read using a portable reader (RIDA^®^- QUICK SCAN II, R-Biopharm AG, Darmstadt, Germany). The RIDA^®^QUICK ADM Monitoring allows quantification of ADM in the 0.5–25.0 μg/mL range [29].

#### 2.4.2. Traditional Detection with ELISA

ADM concentrations in capillary VAMS samples, venous VAMS samples and serum were determined using an in-house developed ADM ELISA with a lower limit of quantification of 0.1 μg/mL [30]. Briefly, MA-ADM28B8 (4 μg/mL) was coated to 96-well plates at 4 °C for 72 h. Serum and VAMS extracts were diluted to a final dilution of 1:2000 in PTAE buffer (phosphate-buffered saline (PBS) + 0.1% bovine serum albumin (BSA) + 0.002% Tween 80 + EDTA), applied on the plate and incubated overnight at 4 °C. The next day, horseradish peroxide (HRP)-conjugated MA-ADM40D8 was applied for the detection of bound ADM and incubated for 2 h at room temperature. Plates were washed and developed using o-phenylenediamine and H_2_O_2_ in citrate buffer and the reaction was stopped with H_2_SO_4_ (4 M). The absorbance was then measured at 492 nm with an ELx808 Absorbance Microplate Reader (BioTek Instrumens Inc., Winooski, VT, USA).

### 2.5. Statistical Analysis

Correlations and agreements between the results obtained by LFT and ELISA were assessed using the Pearson r correlation coefficient, simple linear regression, and Bland–Altman analysis. A two-tailed *p*-value < 0.05 was considered significant. All statistics were carried out using the statistical programs Graphpad Prism 8.3.0 (Graphpad software, San Diego, CA, USA) and IBM SPSS Statistics 25 (IBM SPSS, Costa Mesa, CA, USA).

## 3. Results

### 3.1. Demographics of Study Cohort

Until present, the SUPRA-A trial recruited a total of 10 patients, of whom 6 were randomized (1:1) in the intervention arm. With the exception of one patient, all patients were male. The cohort had a mean age of 54.2 years, with a mean disease duration of 30.5 years. Treatment duration with ADM ranged from 3.9 years to 12.8 years, with a mean psoriasis area and severity index (PASI) at start of therapy of 8.2. Before randomization, all patients were screened with ELISA for supratherapeutic ADM serum trough concentrations (>8 μg/mL). Patients were randomized if on two out of three time points, supratherapeutic trough levels were measured. Overall, patients had a mean ADM concentration of 9.6 μg/mL during screening, ranging from 7.6 to 13.1 μg/mL.

Seven participants agreed to be included in the substudy for microsampling. All patients were highly educated Caucasian men with an average age of 50.3 years. The range of ADM serum trough concentrations obtained in the screening of this subcohort was the same as the one mentioned above, with a mean of 9.7 μg/mL; five participants were randomized to the intervention group.

### 3.2. Rapid Testing of Adalimumab Is Feasible and Valuable in Clinical Setting

**Figure 3:**
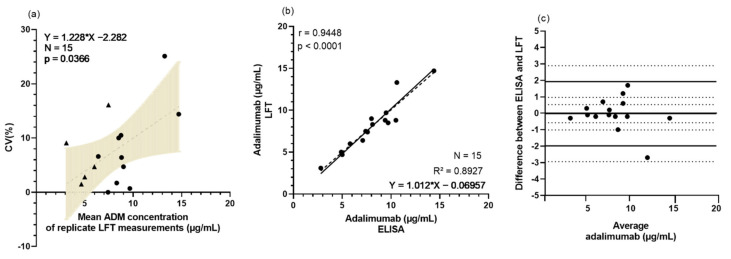
Comparison between LFT and ELISA for quantification of ADM in patient samples from SUPRA-A trial. Serum was collected during ADM maintenance therapy from patients participating in the SUPRA-A trial and drug concentrations were quantified in parallel by two detection methods in duplicate, LFT and ELISA. (**a**) Pearson correlation between the CV (%) and the mean ADM concentrations (μg/mL) measured with LFT by 2 independent researchers. Week 0 and week 13 values are represented by circles and triangles, respectively. The grey range indicates the 95% CI of the best-fit line (simple linear regression). (**b**) Pearson correlation between ADM serum concentrations measured with LFT (*Y*-axis) and in-house sandwich ELISA (*X*-axis). (**c**) Bland–Altman plot for ADM quantification by LFT and with in-house sandwich ELISA. Mean bias and limits of agreement are represented by full lines, 95% CI by dotted lines. Abbreviations: ADM—adalimumab; CI—confidence interval; CV—Coefficient of variation; ELISA—enzyme-linked immunosorbent assay; LFT—lateral flow test; N—number of samples.

A total of 15 samples were collected from 10 patients on week 0 and from 5 patients on week 13. One patient in the control arm had dropped out before the sampling of week 13, hence the missing time point. Lateral flow testing was performed independently by two researchers and showed a mean coefficient of variation (CV) of 7.6% (median: 6.4%; range: 0.0–25.1%), which is slightly lower than the interassay precision reported by the manufacturer [29]. The ADM concentrations in these serum samples ranged from 2.9 to 16.2 μg/mL (Table S1). Based on simple linear regression, a significant concentration-dependent variation of the LFT measurements was observed (Figure 3a).

Next, LFT-obtained ADM concentrations (the mean of replicate measurements for both assessments was used) were compared to ELISA-based quantification. The results showed a good agreement with the reference assay, as indicated by a correlation coefficient R^2^ of 0.89 (Figure 3b), which is somewhat lower than the correlation coefficient (R^2^ = 0.95) previously reported by the manufacturer [29]. The Bland–Altman analysis yielded a mean bias of −0.03 μg/mL, indicating the absence of a significant systematic bias between LFT and ELISA (Figure 3c). Noteworthy, the span covered by the upper and lower limits of agreement (LoA) (−1.98–1.93 μg/mL) is rather big, given the relative narrow therapeutic window of ADM, i.e., 3.51–7.0 μg/mL. This may influence clinical decision-making as categorization of patients as sub- or supratherapeutic may differ, depending on the method of analysis used. However, relatively limited number of samples were included at this point in time—future inclusion of more patients will help to further substantiate this finding.

### 3.3. VAMS Is Suitable for Extraction of Adalimumab

For validation of the VAMS extraction protocol, the extraction efficiency of ADM from VAMS tips was evaluated by spiking the blood of healthy volunteers with known ADM concentrations, ranging from 0 to 20 μg/mL. Extracts were measured 5 times on different days with an in-house ADM ELISA and a mean extraction efficiency was obtained of 114.4% (SD 13.9%, CV 12.2%).

### 3.4. Feasibility of Home Sampling by Non-Experienced Patients Using VAMS

Until present, seven patients were included, resulting in 136 capillary VAMS samples (68 replicates; 4 missing), 40 venous VAMS samples (20 replicates; 2 missing) and 19 serum samples (2 missing) which were eligible for ADM quantification. Ten capillary VAMS samples were excluded from analysis after visual inspection due to undersampling (7.4%).

**Figure 4:**
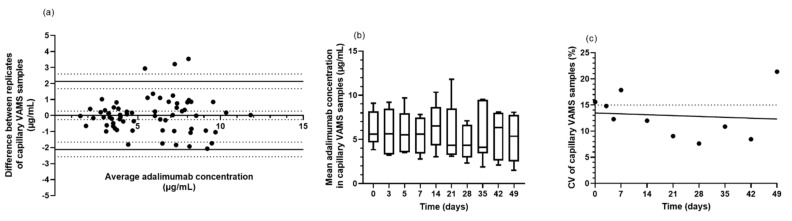
Patients’ performance on microsampling (capillary VAMS). Participants self-sampled VAMS twice in a row, these replicates being taken at 10 time points between day 0 and day 49. ADM was measured in capillary VAMS extracts with an in-house ELISA. (**a**) Bland–Altman plot for ADM quantification in capillary VAMS (n = 65). Mean bias and limits of agreement are represented by full lines, 95% CI by dotted lines. (**b**) Boxplots of mean ADM concentrations in capillary VAMS samples, taken at time points between day 0 and day 49. (**c**) CV (%) of replicates from capillary VAMS samples at the sampling time points between day 0 and day 49. The CV (%) threshold of 15% and the best fit curve (simple linear regression) are indicated by a dotted and full line, respectively. Abbreviations: ADM—adalimumab; CI—confidence interval; CV—Coefficient of variation; ELISA—enzyme-linked immunosorbent assay; N—number of samples; VAMS—volumetric absorptive microsampling.

First, the quality of the patient sampling technique was assessed by comparing biological replicates of capillary VAMS. The difference between the replicates was determined (n = 65) and the CV was calculated. The CV obtained for capillary VAMS samples was 13.5%, which was slightly, though significantly higher than the CV of 7.5% observed for venous VAMS samples (Chi2-test, α = 0.05). The latter were prepared by a trained nurse, dipping the VAMS tips in EDTA-anticoagulated venous whole blood. Overall, a very good correlation was observed between biological replicates of the capillary VAMS samples, with no concentration- or time-dependent variation in the imprecision of home sampling (Figure 4a–c).

**Figure 5:**
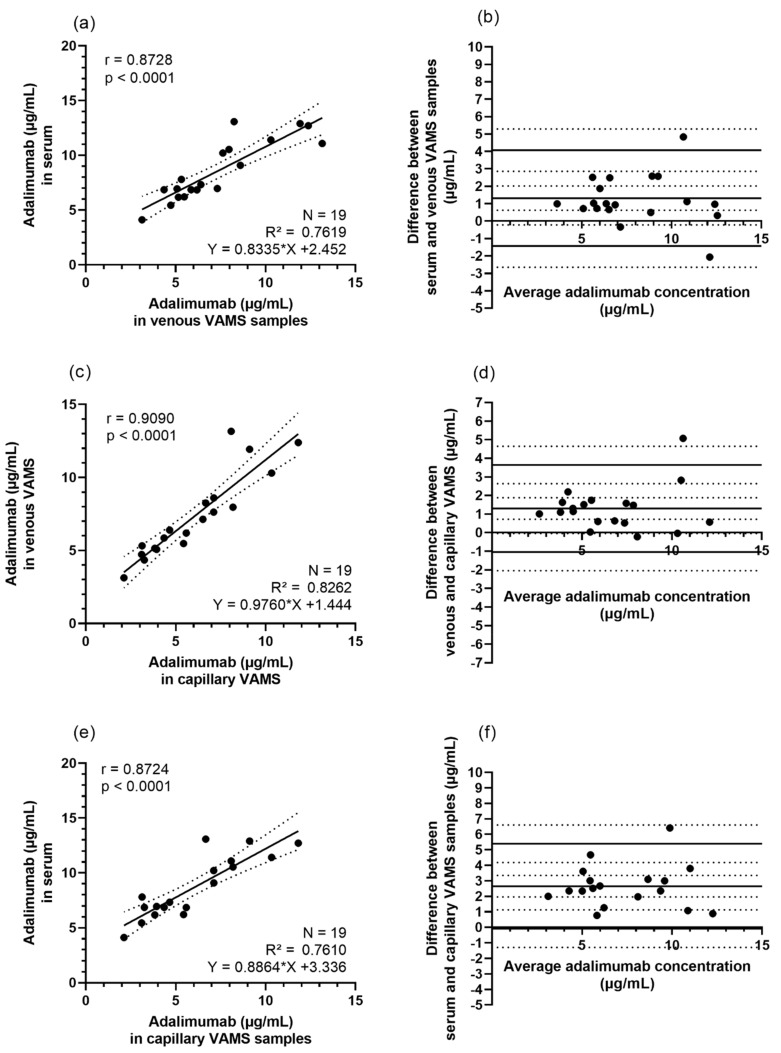
Concordance between capillary VAMS samples, venous VAMS samples and serum (gold standard). (**a**) Pearson correlation of ADM concentrations obtained from venous VAMS samples prepared by nurse (*X*-axis) and serum (*Y*-axis). (**b**) Bland–Altman comparison of ADM concentrations in venous VAMS samples and serum. Mean bias and limits of agreement are represented by full lines, 95% CI by dotted lines. (**c**) Pearson correlation of ADM concentrations obtained from mean venous VAMS samples, prepared by a trained nurse (*Y*-axis) and mean capillary VAMS samples, collected by the patients (*X*-axis). (**d**) Bland–Altman comparison of ADM concentrations in samples obtained by nurse versus patients. Mean bias and limits of agreement are represented by full lines, 95% CI by dotted lines. (**e**) Pearson correlation of ADM concentrations obtained from capillary VAMS samples collected by patients (*X*-axis) and serum (*Y*-axis). (**f**) Bland–Altman comparison of ADM concentrations in capillary VAMS samples and serum. Mean bias and limits of agreement are represented by full lines, 95% CI by dotted lines. Abbreviations: ADM—adalimumab; CI—confidence interval; N—number of samples; VAMS—volumetric absorptive microsampling.

Next, we evaluated how quantification of ADM obtained with VAMS would compare to simultaneously collected serum (gold standard). Hereto, we first compared ADM concentrations obtained from venous VAMS samples (Figure 5a,b) with those obtained from the corresponding serum samples. A good correlation between ADM concentrations in venous VAMS samples [7.32 ± 2.78; 6.92 (5.24–8.63)] and in serum [8.56 ± 2.7; 8.4 (7.3–10.5)] was observed, with a Pearson correlation of 0.87 [mean ± SD; median (IQR), μg/mL].

Then, we examined the concordance of the results obtained from capillary VAMS samples (obtained by patient self-sampling) and simultaneously collected venous VAMS samples. Figure 5c depicts a Pearson correlation of r = 0.91. From the Bland–Altman analysis, a mean significant bias of 1.30 μg/mL was apparent, with 95% CI 0.72–1.87 (Figure 5d; *p* = 0.0002). This implies that ADM concentrations in venous VAMS samples are overall slightly higher than in capillary VAMS samples, collected at the same time point. This capillary venous difference illustrates the need for the application of a capillary-venous correction factor. Based on our cohort, a capillary-venous correction factor of 1.28 was found.

**Figure 6:**
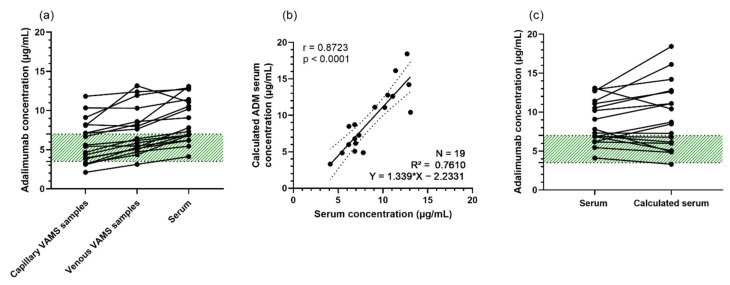
Concordance between capillary VAMS samples, venous VAMS samples and calculated serum (based on a blood-serum and capillary-venous correction factor). (**a**) ADM (μg/mL) in the three matrices, capillary VAMS samples, venous VAMS samples and serum. Green range represents the therapeutic window of ADM (3.51–7.0 μg/mL [8,9]) (**b**) Pearson correlation of ADM concentrations obtained from serum samples (*X*-axis) and calculated ADM serum concentrations based on a blood-serum correction factor of 1.22 and a capillary correction factor of 1.28 (*Y*-axis). (**c**) ADM (μg/mL) in serum (gold standard) and calculated ADM serum concentration, based on a blood-serum correction factor of 1.22 and a capillary-venous correction factor of 1.28. Green range represents the therapeutic window of ADM (3.51–7.0 μg/mL [8,9]). Abbreviations: ADM—adalimumab; CI—confidence interval; N—number of samples; VAMS—volumetric absorptive microsampling.

Finally, we compared ADM concentrations obtained from capillary VAMS samples with those obtained from the corresponding serum samples. As shown in Figure 5 (panel e,f), a good correlation between ADM concentrations in capillary VAMS samples [5.67 ± 2.47; 5.48 (3.52–7.61)] and in serum [8.56 ± 2.7; 8.4 (7.3–10.5)] was observed, with a Pearson correlation of 0.87 [mean ± SD; median (IQR), μg/mL]. A significant mean bias of 2.7 μg/mL (95% CI: 1.96, 3.35) was found by Bland–Altman analysis (*p* < 0.0001), implying that lower concentrations are obtained from VAMS samples, compared to the corresponding serum samples, which can be expected, as ADM resides in the serum/plasma fraction of blood. By multiplying the ADM concentration from capillary VAMS samples with the capillary-venous and blood-serum correction factors of, respectively, 1.28 and 1.22, the calculated ADM serum concentration was determined (Figure 6). As depicted in Figure 6c, this may be an overcorrection due to the small sample size of this cohort.

### 3.5. Patient Experience with VAMS

All participants completed the substudy according to the protocol, which by itself was already considered to be a success regarding acceptance of VAMS as a collection technique. All samples, shipped by regular mail, were received within an acceptable time frame (on average 5 days, range [3,4,5,6,7,8]).

Next, we used a questionnaire to evaluate the patients’ perception on self-sampling at home based on Van Uytfanghe et al. and Mbughuni et al., with minor adaptations [28,31]. The questionnaire consisted of 10 main questions and gauged about the clarity of instructions, experience, performance, user-friendliness, acceptability/pain and preference.

**Figure 7:**
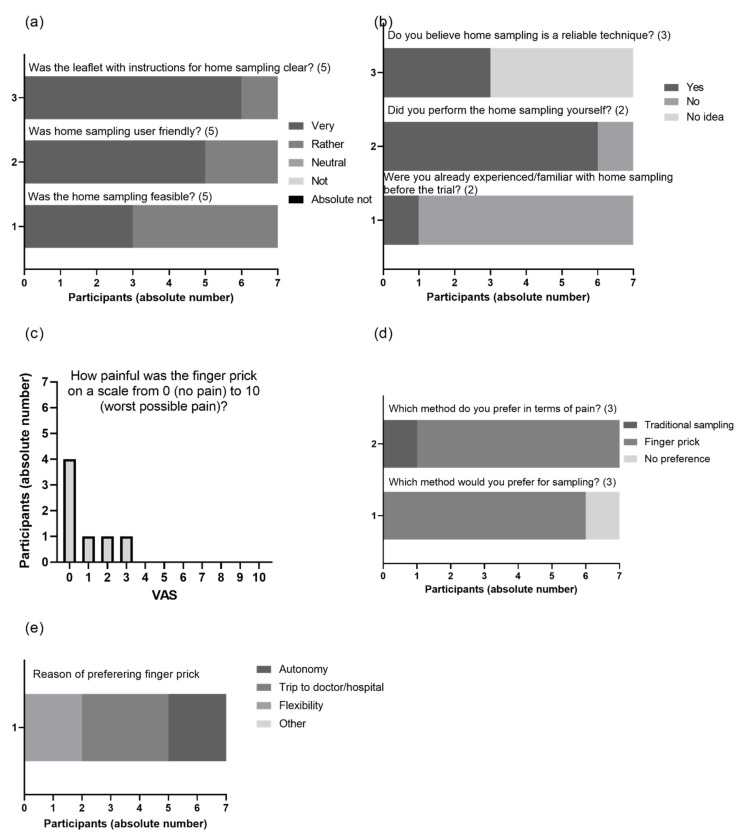
Microsampling was found to be easily executed by patients and preferred over venous sampling. After completing the substudy for microsampling, participants were sent an electronic survey to inquire into their experience. Patients scored questions on execution (**a**) and execution experience (**b**). Pain was quantified (**c**,**d**) and compared to traditional sampling (**d**,**e**). n = 7 The number between brackets in panel (**a**,**b**,**d**) indicates the number of possible answers.

All patients (n = 7) completed the survey. Five out of seven participants (71.4%) had a higher degree (bachelor or master). Except one, none of the respondents had experience with performing VAMS before. All patients judged the clarity of the instructions to be clear or very clear and evaluated the performance as very or rather user-friendly and feasible (Figure 7a). None of the patients felt the need to consult for additional information (data not shown). All patients self-sampled, except for one participant, who stated their partner was a nurse who performed the finger prick (Figure 7b). On a scale of 1 (not painful at all) to 10 (very painful), participants scored 3 or less, with 60% rating it a zero (Figure 7c). Moreover, with the exception of one patient, all patients scored finger prick sampling as less painful compared to a conventional blood draw (Figure 7d). With a higher degree of flexibility/autonomy and no transportation to the clinic, this translates to the vast majority (85.7%) preferring this type of sampling over a conventional blood draw (Figure 7d,e).

For VAMS to be adopted by patients in the context of TDM in psoriasis, we inquired into patients’ acceptance. All participants scored the technique as user-friendly for use at home (Figure 7a). In addition, all patients agreed that it would be feasible to perform this regularly. When asked about an acceptable frequency to use VAMS, a monthly basis had the most votes (data not shown). Although patients were positive about this sampling technique, more than half of the patients was uncertain about the reliability of the sampling technique (Figure 7b), reflecting the gap of knowledge.

## 4. Discussion

Although the use of TDM in psoriasis is still not standard practice, parallel research into enabling tools is required to facilitate implementation. As Figure 1 illustrates, TDM requires various steps, and especially the first step, encompassing sampling, will pose most challenges regarding implementation. Easy and feasible sampling is a great challenge, with a healthcare professional being required for a traditional blood draw. Furthermore, serum preparation from whole blood requires the sample to be handled in a laboratory setting.

Here, we investigated the home-based use of VAMS by psoriasis patients treated with ADM for drug quantification. We focused on both the technical performance and the patient’s user experience. From a technical perspective, our extraction protocol showed a satisfactory extraction efficiency, with adequate reproducibility. As TDM is based on narrow therapeutic windows, a robust extraction is essential to ensure appropriate interpretation and treatment management plan. Patients’ performance assessed by replicates was deemed acceptable, with a CV of 13.5%. As no long-term data were collected, at this point no conclusions can be drawn related to ‘performance fatigue’. Performance compared to a trained nurse also showed satisfactory results with a slightly, though significantly higher CV than the CV derived under controlled circumstances for sampling, 7.5%. Based on this, it could be estimated that home sampling accounted for 11.2% of the total imprecision of the method.

In addition, a good correlation between the three matrices, capillary VAMS samples, venous VAMS samples and serum was obtained. At this point it is too early to make definitive statements on whether capillary samples can yield data that can reliably steer dose adaptations (this will become clear in the ongoing trial and was beyond the scope of the current pilot study). However, the data so far indicate that 2 kinds of corrections are required to predict serum concentrations based on capillary blood concentrations: a capillary-venous correction of 1.28, and a blood-serum correction of 1.22. The latter is obvious, as ADM resides in the serum fraction of blood, and 20 μL of (dried) blood only contains ~12 μL of serum (in the case of ~40% haematocrit blood). However, more data are needed to validate these correction factors and verify these on independent sample sets. In future, these data will provide further insight into the suitability of VAMS as an adequate substitute for conventional sampling for ADM TDM.

Besides the technical suitability of microsampling for TDM of ADM in psoriasis patients, it is also important to acknowledge the patients’ perception, as they are the end users. Based on our questionnaire, it can be concluded that the patients were overall positive about home-based microsampling. Even more, participants in this pilot study preferred this type of sampling over a conventional blood draw, but this needs to be confirmed in a larger cohort. This is in agreement with results obtained by Morgan et al., who showed that 81% of the participants preferred VAMS collection, and is in line with the preference for VAMS in the cohort studied by Verougstraete et al. [23,32]. Essential to implementation, we also investigated the time it took for a sample to reach our laboratory. Based on our limited data, the time window was deemed acceptable for psoriasis management with ADM. Most biologics are administered from twice a month to every 3 months—rendering an average of 5 transport days (range: 3–8 days) acceptable for the physician to adapt the management plan.

In this paper, we did not address the impact of storage conditions on extraction efficiency. However, we refer to Bloem et al. where a similar technique has been investigated for several storage conditions [17]. In addition, Li et al. obtained very encouraging drug recovery data after short and long term storage for biotherapeutics daclizumab and trastuzumab [33]. Ideally, when patients require a change in management plan, results should become available relatively fast, making long-term storage impact irrelevant. The impact of parameters such as temperature and humidity should be investigated in real world settings for conclusive evidence. To lower the threshold of using VAMS within the patient community and empower patients to monitor their drug concentrations, in the future, microsampling kits could become available at the pharmacy, or could be provided by the treating physician upon treatment with biologics.

After sampling, trough drug concentrations are traditionally measured by ELISA, requiring laboratory equipment (e.g., a shaker and dedicated reader) and are time-consuming. An additional disadvantage of ELISA is the ratio of performance time to sample size. As ELISA is standard-dependent, a single sample run is considered wasteful. To this end, the (complementary) use of immunochromatographic lateral flow testing allows for a more satisfactory ratio of performance time to sample size. The LFT assay investigated here was demonstrated to be applicable in clinical practice, with a turnover time of less than 30 min after serum preparation. Although it was compared to only one ELISA kit for ADM quantification [25,34], our data show acceptable results for clinical applicability. As the therapeutic window of ADM is relatively narrow [8,9], interpretation of results should be done taking into account the type of assay. In the limited dataset from this study, a good agreement was found between LFT and ELISA, suggesting that no adaptation of the therapeutic window would be required when implementing this LFT assay—obviously, more samples are required to further substantiate this finding. The current LFT assay was performed on serum samples, and its compatibility with VAMS remains to be elucidated. Ultimately, a combination of both tools would truly enable rapid and easy monitoring.

Limitations of this pilot study inherently include the small sample size for both microsampling and rapid testing. In addition, we did not address various conditions to assess the impact on both tools. For instance, we currently lack data on storage conditions and how ADM concentrations in VAMS samples are affected. All LFT measurements were executed by trained lab personnel. As all measurements were performed in an academic hospital setting, extrapolation to private practices is limited. It should also be noted that the used LFT cassettes are not compatible with all LFT-readers.

Strengths of this study reside in the affinity with real world evidence as SUPRA-A is considered a pragmatic trial, in addition to the use of public postal services for sample transport. Furthermore, participants had access to adequate educational material to execute microsampling. Lastly, in addition to laboratory evaluations, we also considered and investigated the study participants as end users—rendering this study comprehensive.

## 5. Conclusions

Microsampling for ADM TDM in the context of psoriasis treatment is a valuable alternative to traditional blood sampling, enabling patient-centric TDM. VAMS, as applied here, can be performed by non-experienced patients at home, potentially allowing to reach a greater patient community. In addition, rapid testing by LFT of ADM allows the dermatologist to rapidly obtain results that may impact a patient’s treatment plan.

The results presented here provide preliminary evidence, revealing that LFT and microsampling are promising tools to facilitate TDM of ADM in clinical practice. Though the data is from a limited sample size, both tools pose interesting fields of further investigation for TDM in psoriasis.

## Data Availability

The data presented in this study can be found at https://osf.io/2bk7u hosted at Open Science Framework and access can be obtained upon request.

## Supplementary Materials

The following supporting information can be downloaded at: https://www.mdpi.com/article/10.3390/jcm11113011/s1, Figure S1: Written instruction (in Dutch) for patients for home sampling by use of VAMS after finger prick; Table S1: Lateral Flow Testing results of adalimumab concentrations in SUPRA-A trial participants.
