# Supplementary material for: Promising Tools to Facilitate the Implementation of TDM of Biologics in Clinical Practice
Source: J Clin Med. 2022 May 26;11(11):3011. doi: 10.3390/jcm11113011 (PMC9181069; doi:10.3390/jcm11113011)
Supplement: Supplementary file 1 [file jcm-11-03011-s001.zip › jcm-1732643-supplementary.pdf]

Supplementary Figure S1. Written instruction (in Dutch) for patients for home sampling by use of VAMS after finger prick

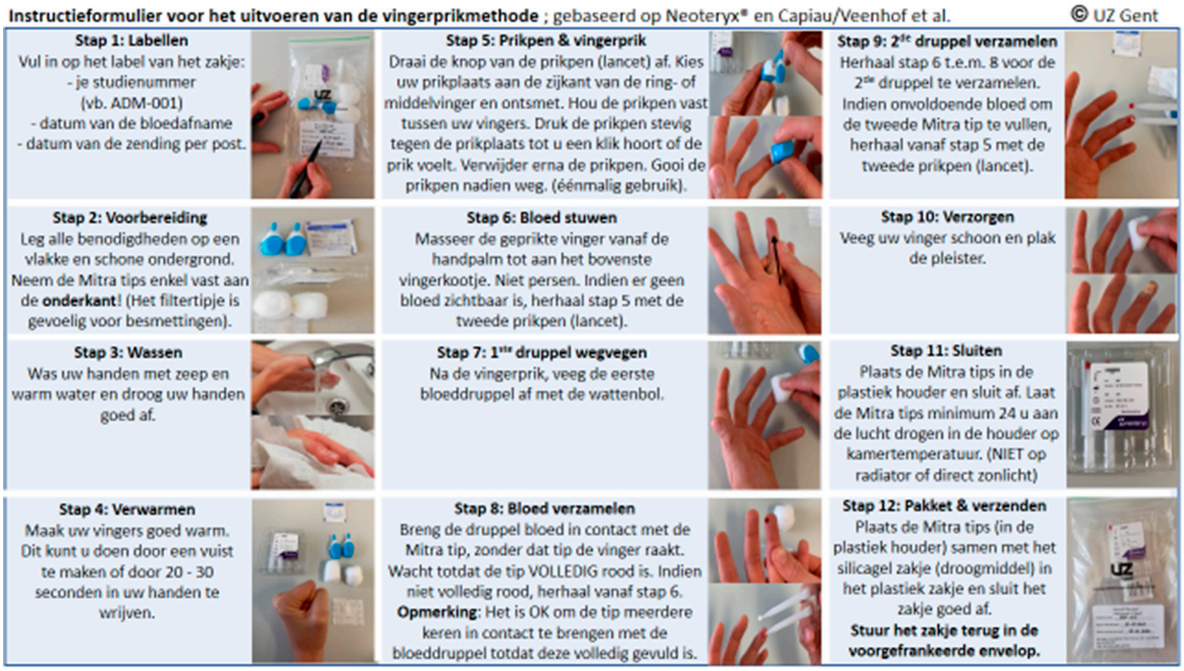

Instructieformulier voor het uitvoeren van de vingerprikmethode ; gebaseerd op Neoteryx® en Capiau/Veenhof et al. © UZ Gent

Stap 1: Labellen
Vul in op het label van het zakje:
- je studienummer (vb. ADM-001)
- datum van de bloedafname
- datum van de zending per post.

Stap 2: Voorbereiding
Leg alle benodigdheden op een vlakke en schone ondergrond. Neem de Mitra tips enkel vast aan de **onderkant**! (Het filtertipje is gevoelig voor besmettingen).

Stap 3: Wassen
Was uw handen met zeep en warm water en droog uw handen goed af.

Stap 4: Verwarmen
Maak uw vingers goed warm. Dit kunt u doen door een vuist te maken of door 20 - 30 seconden in uw handen te wrijven.

Stap 5: Prikpen & vingerprik
Draai de knop van de prikpen (lancet) af. Kies uw prikplaats aan de zijkant van de ring- of middelvinger en ontsmet. Hou de prikpen vast tussen uw vingers. Druk de prikpen stevig tegen de prikplaats tot u een klik hoort of de prik voelt. Verwijder erna de prikpen. Gooi de prikpen nadien weg. (éénmalig gebruik).

Stap 6: Bloed stuwen
Masseer de geprikte vinger vanaf de handpalm tot aan het bovenste vingerkootje. Niet persen. Indien er geen bloed zichtbaar is, herhaal stap 5 met de tweede prikpen (lancet).

Stap 7: 1ste druppel wegvegen
Na de vingerprik, veeg de eerste bloeddruppel af met de wattenbol.

Stap 8: Bloed verzamelen
Breng de druppel bloed in contact met de Mitra tip, zonder dat tip de vinger raakt. Wacht totdat de tip VOLLEDIG rood is. Indien niet volledig rood, herhaal vanaf stap 6.
Opmerking: Het is OK om de tip meerdere keren in contact te brengen met de bloeddruppel totdat deze volledig gevuld is.

Stap 9: 2de druppel verzamelen
Herhaal stap 6 t.e.m. 8 voor de 2de druppel te verzamelen. Indien onvoldoende bloed om de tweede Mitra tip te vullen, herhaal vanaf stap 5 met de tweede prikpen (lancet).

Stap 10: Verzorgen
Veeg uw vinger schoon en plak de pleister.

Stap 11: Sluiten
Plaats de Mitra tips in de plastiek houder en sluit af. Laat de Mitra tips minimum 24 u aan de lucht drogen in de houder op kamertemperatuur. (NIET op radiator of direct zonlicht)

Stap 12: Pakket & verzenden
Plaats de Mitra tips (in de plastiek houder) samen met het silicagel zakje (droogmiddel) in het plastiek zakje en sluit het zakje goed af.
Stuur het zakje terug in de voorgefrankeerde envelop.

Supplementary Table S1

Table S1. Lateral Flow Testing results of adalimumab concentrations in SUPRA-A trial participants

	Week	Adalimumab (μg/ml) M1	Adalimumab (μg/ml) M2	Mean ± SD	CV (%)
Patient A*	0	7.4	7.4	7.4 ± 0.0	0.0
	13	5.1	4.9	5.0 ± 0.1	2.8
Patient B	0	9.7	9.6	9.7 ± 0.1	0.7
Patient C	0	8.1	9.4	8.8 ± 0.9	10.5
Patient D*	0	13.2	16.2	14.7 ± 2.1	14.4
	13	8.3	6.6	7.5 ± 1.2	16.1
Patient E*	0	8.4	8.2	8.3 ± 0.1	1.7
	13	2.9	3.3	3.1 ±0.3	9.1
Patient F*	0	15.6	10.9	13.3 ± 3.3	25.1
	13	4.7	4.6	4.7 ± 0.1	1.5
Patient G	0	6.1	6.7	6.4 ± 0.4	6.6
Patient H*	0	9.1	7.9	8.5 ± 0.8	10.0
	13	5.8	6.2	6.0 ± 0.3	4.7
Patient I*	0	9.3	8.7	9.0 ± 0.4	4.7
	13	NA	NA	NA	NA
Patient J	0	9.2	8.4	8.8 ± 0.6	6.4

Abbreviations and symbols: M1,2 - measurement; SD - standard deviation; CV - coefficient of variation; *participant randomized to dose reduction arm; NA – Sample not available due to drop-out before sampling timepoint.
